# COVID-19 pneumonia: current evidence of chest imaging features, evolution and prognosis

**DOI:** 10.1007/s42058-021-00068-0

**Published:** 2021-05-04

**Authors:** Anna Rita Larici, Giuseppe Cicchetti, Riccardo Marano, Lorenzo Bonomo, Maria Luigia Storto

**Affiliations:** 1 Department of Radiological and Hematological Sciences, Catholic University of the Sacred Heart, Rome, Italy; 2 Department of Diagnostic Imaging, Oncological Radiotherapy, and Hematology, Fondazione Policlinico Universitario “A. Gemelli” IRCCS, Rome, Italy; 3 Bracco Diagnostics Inc., Global Medical and Regulatory Affairs, Monroe Twp, NJ USA

**Keywords:** COVID-19 pneumonia, Imaging findings, Evolution, Prognosis, Chest X-ray, High-resolution computed tomography

## Abstract

COVID-19 pneumonia represents a global threatening disease, especially in severe cases. Chest imaging, with X-ray and high-resolution computed tomography (HRCT), plays an important role in the initial evaluation and follow-up of patients with COVID-19 pneumonia. Chest imaging can also help in assessing disease severity and in predicting patient’s outcome, either as an independent factor or in combination with clinical and laboratory features. This review highlights the current knowledge of imaging features of COVID-19 pneumonia and their temporal evolution over time, and provides recent evidences on the role of chest imaging in the prognostic assessment of the disease.

## Introduction

Since December 2019, a novel coronavirus, the SARS-CoV-2 (Severe Acute Respiratory Syndrome Coronavirus 2) has spread across the globe [1].

Despite a potential multi-systemic involvement, lung is by far the organ most commonly involved by SARS-CoV-2 infection [2]. The so-called coronavirus disease 2019 (COVID-19) pneumonia may present various degrees of severity and respiratory impairment, ranging from mild cases with flu-like symptoms to severe forms with acute respiratory distress syndrome (ARDS), requiring intensive care support [3]. Mortality rates of about 40% have been reported in the latter cases [4].

Chest X-ray (CXR) and high-resolution computed tomography (HRCT) provide a fundamental contribute in the diagnostic work-up and management of patients affected by COVID-19 pneumonia. Indeed, apart from diagnostic purposes [5], imaging plays a key role in the assessment of disease burden and evolution over time. CXR is particularly helpful in the serial evaluation of hospitalized patients, especially in those requiring intensive care unit (ICU) admission [6]. On the other hand, HRCT is the modality of choice in providing prognostic information in patients with COVID-19 pneumonia [7] and in assessing eventual complications [8–10].

Due to the predisposition to thrombotic and thromboembolic events caused by SARS-CoV-2 [11, 12], the association between COVID-19 pneumonia and the occurrence of acute pulmonary embolism (PE) has been recently documented in a non-negligible number of patients [13, 14]. Hence, the use of CT pulmonary angiography (CTPA) might be advised, when clinically appropriate.

The aim of this review is to depict the evolution over time of COVID-19 pneumonia findings, from the acute to the late phase, and to underline the role of thoracic imaging in the prognostic evaluation of this global threatening disease.

## Phases of COVID-19 pneumonia

Autoptic specimens of deceased patients with COVID-19 pneumonia revealed the presence of diffuse alveolar damage (DAD) at different stages as the major pathologic finding [15–17]. DAD tends to evolve through three progressive phases [18]. The first phase is the *exudative phase*, characterized by capillary congestion and edema, alveolar haemorrhage, and hyaline membrane formation. Approximately 7 days after the start of injury, a proliferative phase occurs, consisting of fibroblast proliferation within the interstitium and alveoli, thickening of the interlobular septa, type 2 pneumocyte hyperplasia**,** parenchymal remodelling and appearance of organizing pneumonia (OP) foci. Finally, after 2 weeks, the fibrotic phase may develop, with collagen deposition and progressive fibrotic changes [18]. Each phase is associated with different imaging findings, which correlate well with the progression of DAD over time [19]. These phases correspond to the evolution of imaging features in COVID-19 pneumonia.

Pan et al. identified on HRCT four consecutive temporal stages in patients who recovered from COVID-19 pneumonia: the early stage (0–4 days from the onset of symptoms), the progressive stage (5–8 days), the peak stage (9–13 days), and the absorption stage (≥ 14 days) [20]. In another study by Wang et al. a similar time course was described, even though a delayed dissipation phase was noticed, possibly due to the inclusion of more severe cases [21]. Comparable evolution of radiological findings has also been reported on CXR [22, 23].

With a certain overlap, each stage is characterized by different imaging features and degrees of lung involvement. At first, a progression in terms of density, number and extension of lung abnormalities is observed [21, 24], followed by a gradual resolution of the findings in cases of favourable disease course. In the late phase, possible residual fibrotic abnormalities or development of progressive interstitial fibrosis might be observed [25, 26].

Some authors have recognized different patterns of the temporal evolution of radiological abnormalities [27, 28]. In this regard, Shi et al. identified four different patterns of longitudinal evolution on CT scans in patients with variable disease severity [27]. *Type 1* showed an initial progression of the abnormalities till a peak level, followed by progressive improvement, and this was the most common pattern (46% of patients); *type 2* characterized by a progressive radiological deterioration despite medical treatment (32% of cases); *type 3* demonstrated a progressive radiological improvement over time and was found in 14% of patients. The last pattern (*type 4*), showing a stable radiological appearance over time, was observed in a minority of patients (9%). Interestingly, type 2 was associated with a poorer prognosis when compared with type 1 and 3 [27]. Likewise, Han et al. analyzed longitudinal changes on CT for 4 weeks since hospital admission in patients with favorable course of disease, observing similar patterns of radiological evolution [28]; the dynamic evolution of chest CT manifestations have been recently confirmed by a meta-analysis by Zhou at el. [29].

## Imaging findings of COVID-19 pneumonia by phase

### Early phase

At the beginning of the disease, the main imaging findings on HRCT are ground-glass (GG) opacities with a typical bilateral and multilobar distribution and middle-lower lung predominance, mostly located in the subpleural and dorsal regions of the lungs [30]. This appearance has been described in the large majority of patients, with a prevalence of 83.3% and 81%, respectively, according to two different systematic reviews and meta-analyses [31, 32]. Less commonly, a mixed pattern of consolidation and GG opacities can be observed [30]. Another possible presentation in this phase is a unilateral distribution of lung abnormalities, with focal or few patchy unilobar rounded GG opacities [6, 27], which has been described in 15% of patients with COVID-19 pneumonia according to a meta-analysis including 3.466 patients [32].

On CXR early lung involvement demonstrates a predominance of reticular opacities, which progressively become hazy confluent GG opacities [23].

**Fig. 1:**
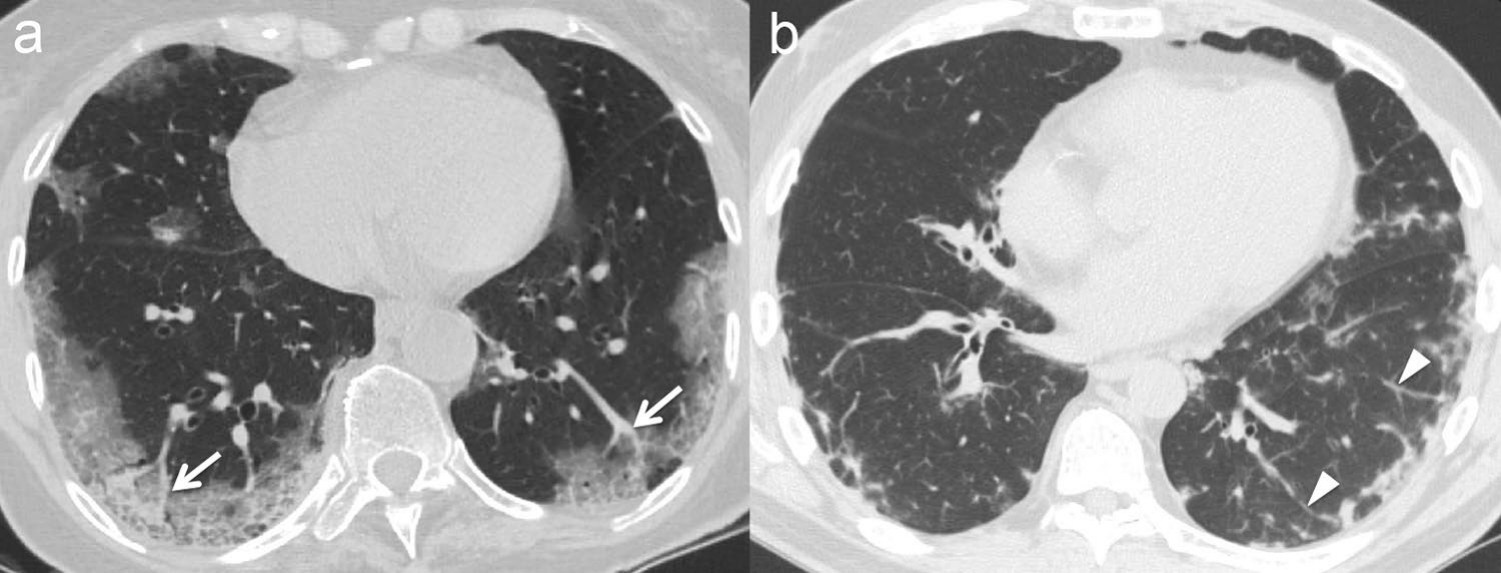
**a** Patient with COVID-19 pneumonia. The CT scan shows a crazy-paving pattern with typical predominant subpleural and bilateral distribution at the lung bases; note the presence of "enlarging vessel" sign within the areas of increased lung density (arrows). **b** Patient with H1N1 influenza-associated pneumonia. The CT scan shows multifocal small areas of consolidation and perilobular opacities with bilateral subpleural distribution at the lung bases, associated with some GG areas in the left lower lobe; note the normal caliber of pulmonary vessels in the proximity of the pulmonary abnormalities (arrowheads)

A typical finding of the early stage of COVID-19 pneumonia described on HRCT is the evidence of subsegmental vessel enlargement (> 3 mm diameter) within the areas of GG [33–37]. This sign is possibly related to hyperaemia and vessel wall injury induced by inflammation [34]. Indeed, lung damage caused by SARS-CoV-2 infection is linked to the development of a “cytokine storm”, which can cause lung endothelial cells injury and apoptosis [38], ultimately leading to diffuse endothelial damage and the development of vascular microthrombosis [17]. Described in more than 80% of cases in some cohorts [35, 37] and with a pooled prevalence of 72.9% according to Adams et al. [32], this sign may help to differentiate COVID-19 from other viral pneumonia [39] (Fig. 1). Later in the progressive phase, as consolidation replaces the areas of GG, this sign becomes barely visible due to the obscuration of the pulmonary vasculature, while during absorption vessel caliber returns normal. Therefore, vessel enlargement can be used not only to assess the diagnosis of COVID-19 pneumonia, but also as a marker of the early stage of disease.

It has to be considered that, in the early phase, imaging can be negative even in symptomatic subjects [40]. When assessing patients by the time of symptoms onset, Bernheim et al. reported a 56% rate of false negative (FN) on HRCT between 0 and 2 days and noted a progressive rate decline of FN over time (9% at 3–5 days and 4% at 6–12 days) [41]. The same decreasing trend of FN has been reported for CXR, from 36.7% (0–2 days from the onset of symptoms) to 16.1% (after 9 days) [23].

### Progressive and peak phases

As described in a recently published systematic review [24], the progressive phase is associated with an increase in size and number of lung parenchymal abnormalities, leading to an overall rise of the total disease extent [21, 42].

**Fig. 2:**
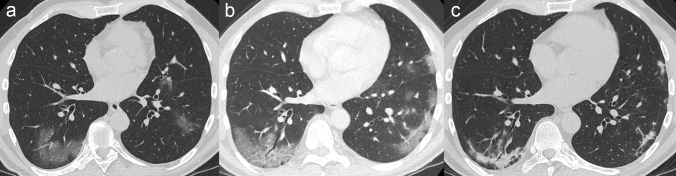
CT scans of a patient with COVID-19 pneumonia demonstrating the typical evolution of the disease by phases. **a** In the early phase, GG opacities are the predominant finding. **b** 10 days after the onset of symptoms (progressive to peak phase) more extensive GG areas with crazy paving appearance and typical distribution at the lung periphery are observed. **c** One week later, GG areas decline and lead to the appearance of multifocal consolidation with mild parenchymal distortion

Particularly, GG opacities increase in size, with a possible diffuse distribution of the lesions. Smooth interlobular septal thickening can appear within the GG areas, leading to a *crazy paving* pattern on HRCT [30], which has been reported in up to 34.9% of patients [32]. At the same time, GG opacities progressively turn into consolidation, thus determining the development of a mixed pattern with a combination of consolidative and GG areas [21, 24] (Fig. 2).

The presence of hyperlucent areas within the consolidation has been described on HRCT during the progressive phase and has been indicated as the “*vacuolar*” *sign*. This finding is possibly caused by the inhomogeneous involvement and/or by the incomplete filling of the alveoli by edema and fibrinous exudates [43].

All the abovementioned HRCT features continue to progress up to day 9–13 of illness, reaching a peak of lung involvement around day 10, as reported by some authors who used a semiquantitative CT scoring system to quantify lung involvement [20, 21, 43]. At this time, the extent of GG abnormalities continues to decline, leading to a multifocal consolidative appearance of the lungs [21, 24] (Fig. 2). In this phase, due to parenchymal remodelling, traction bronchiectasis and bronchiolectasis may appear within the consolidation; these findings have been reported with a pooled prevalence of 24.2% [32].

**Fig. 3:**
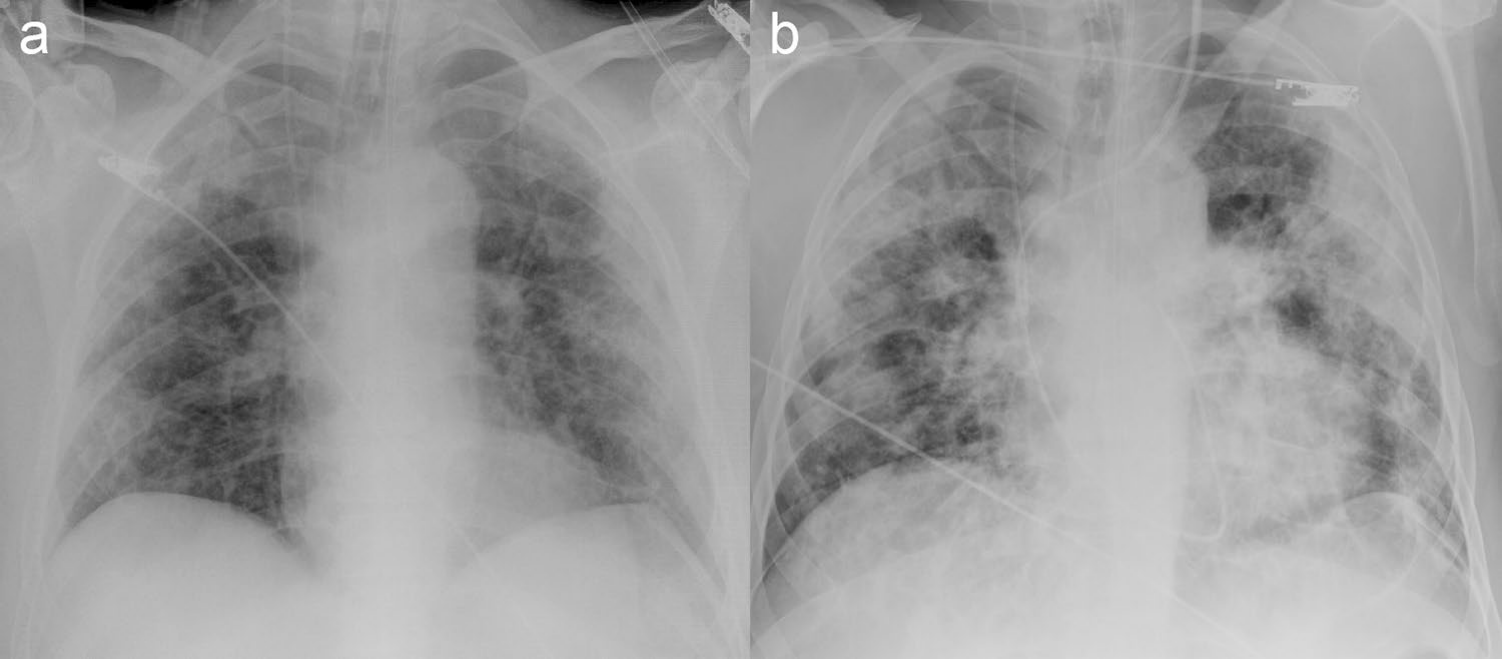
Bedside chest radiograms of a patient with a severe condition of COVID-19 pneumonia showing the temporal lung changes from the early phase (**a**), characterized by bilateral interstitial opacities with peripheral mid-lower lung distribution, to the peak phase (**b**) with bilateral and more extensive confluent alveolar opacities. Note the presence of endotracheal tube and central venous catheter in both radiographs

CXRs show the same temporal evolution as described on HRCT, with a progressive increase of confluent alveolar opacities having a typical predominant peripheral distribution, bilaterally [22, 23] (Fig. 3).

In patients developing an ARDS (up to 40% of COVID-19 pneumonia cases, 20% of whom experiencing a severe form) [44], lung involvement is usually diffuse, and massive bilateral alveolar opacities and consolidation can be seen on CXR and HRCT respectively (“*white lung*” appearance) [45].

In advanced cases, uncommon ancillary findings can be observed, such as pleural or pericardial effusion (usually mild to moderate in extent); these have been described in 5% and 3.6% of patients, respectively, by Ojha et al. [24]. Mediastinal lymph node enlargement can also occur [30].

In this phase, other imaging findings, such as *perilobular opacities*, *subpleural lines* and *reversed halo sign* [46] might become visible on HRCT, usually with patchy bilateral subpleural and peribronchovascolar distribution [30, 41]; in two meta-analyses, the reversed halo sign showed a pooled prevalence of 2.4% and 11.1%, respectively [24, 32]. These features are typically associated with the OP pattern [47], and are an expression of the proliferative phase of DAD occurring in COVID-19 pneumonia [48, 49].

### Absorption phase

Starting from day 14 of illness, a decrease in the extent of lung involvement can be seen [20, 24, 43], which becomes more evident after the 3rd week of illness [43].

In this stage, a progressive reduction in size and number of the abnormalities (consolidation, mixed consolidation and GG and *crazy paving*) is observed, with residual persistence of GG areas [24, 43], which might be misinterpreted as a persistent disease.

**Fig. 4:**
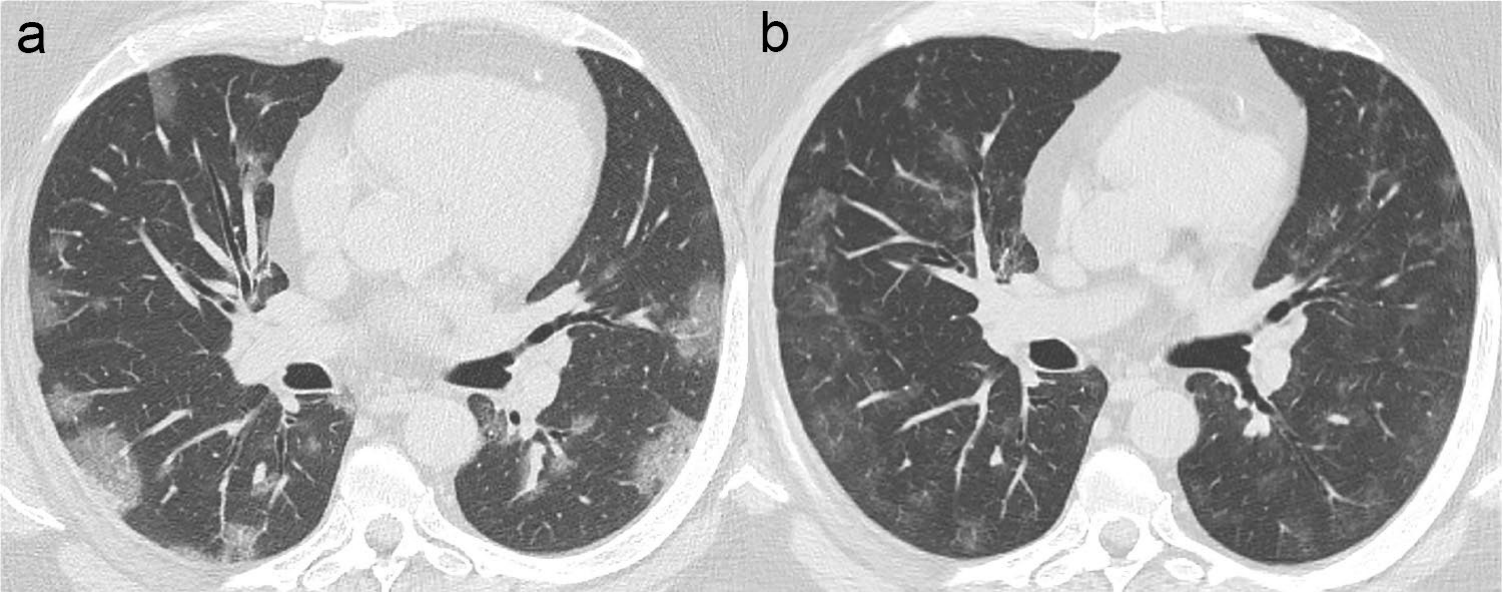
Axial HRCT scans of COVID-19 pneumonia in the absorption phase. Image in **b** nicely demonstrates a decrease in density of the multifocal GG opacities observed in **a**, with a more extensive bilateral lung involvement (“*tinted*” *sign*)

A decrease in density of the consolidation and GG opacities coupled with an increase in the extent of the involved areas has been described as an HRCT sign of absorption (Fig. 4). Defined as “*tinted*” *sign*, this feature might be related to the gradual resolution of the inflammation followed by re-expansion of the alveoli [50].

Other CT features, namely subpleural lines, bronchial distortion, and parenchymal bands, are expressions of parenchymal injury repair. Even though previously evident in some degrees, they become almost constantly visible during this phase [43]. This is probably due to a progressive but asynchronous repair process of the SARS-CoV-2-related lung injury, where lung portions with progressive involvement coexist with other areas characterized by dissipative phenomena [43].

The absorption phase usually shows a long temporal course and can continue even after complete patient recovery and discharge [42, 50, 51] with HRCT findings, such as residual consolidation, faint GG opacities, and bands still evident after 4 weeks from symptoms onset [20, 21]. Residual HRCT findings have been observed in this phase in up to 98.1% of cases, according to Ding et al. [40]. In another cohort of 149 patients undergoing longitudinal HRCT evaluation after recovery [50], only 8.1% showed complete resolution of the abnormalities at the end of hospitalization. On the 3rd week after discharge, still more than 40% of patients demonstrated residual findings, in most of the cases showing GG opacities [50]. In this regard, Huang et al. reported that a prompt treatment is correlated with a more rapid clearance of the abnormalities on HRCT [52].

### Late phase

Despite the removal of the cause of lung injury, the eradication of SARS-CoV-2 does not assure the absence of any long-term sequelae, such as the development of progressive fibrotic abnormalities [25].

Indeed, fibrotic changes, with the almost complete destruction of areas of lung parenchyma, have been demonstrated on pathological specimens of patients deceased because of COVID-19 pneumonia [16].

It is possible that the majority of patients with mild disease (80%) will not be affected by any residual abnormalities, which are conversely expected in patients who experienced severe illness, with the development of ARDS and need for advanced respiratory support [53]. Nevertheless, due to the huge viral spread, even if rare, this late complication may represent a major healthcare issue [25].

After discharge, various degrees of respiratory impairment related to fibrotic abnormalities have been described in almost half of the patients, with 25% showing reduced total lung capacity; this latter was more pronounced in those patients who sustained a severe condition of COVID-19 pneumonia [54].

**Fig. 5:**
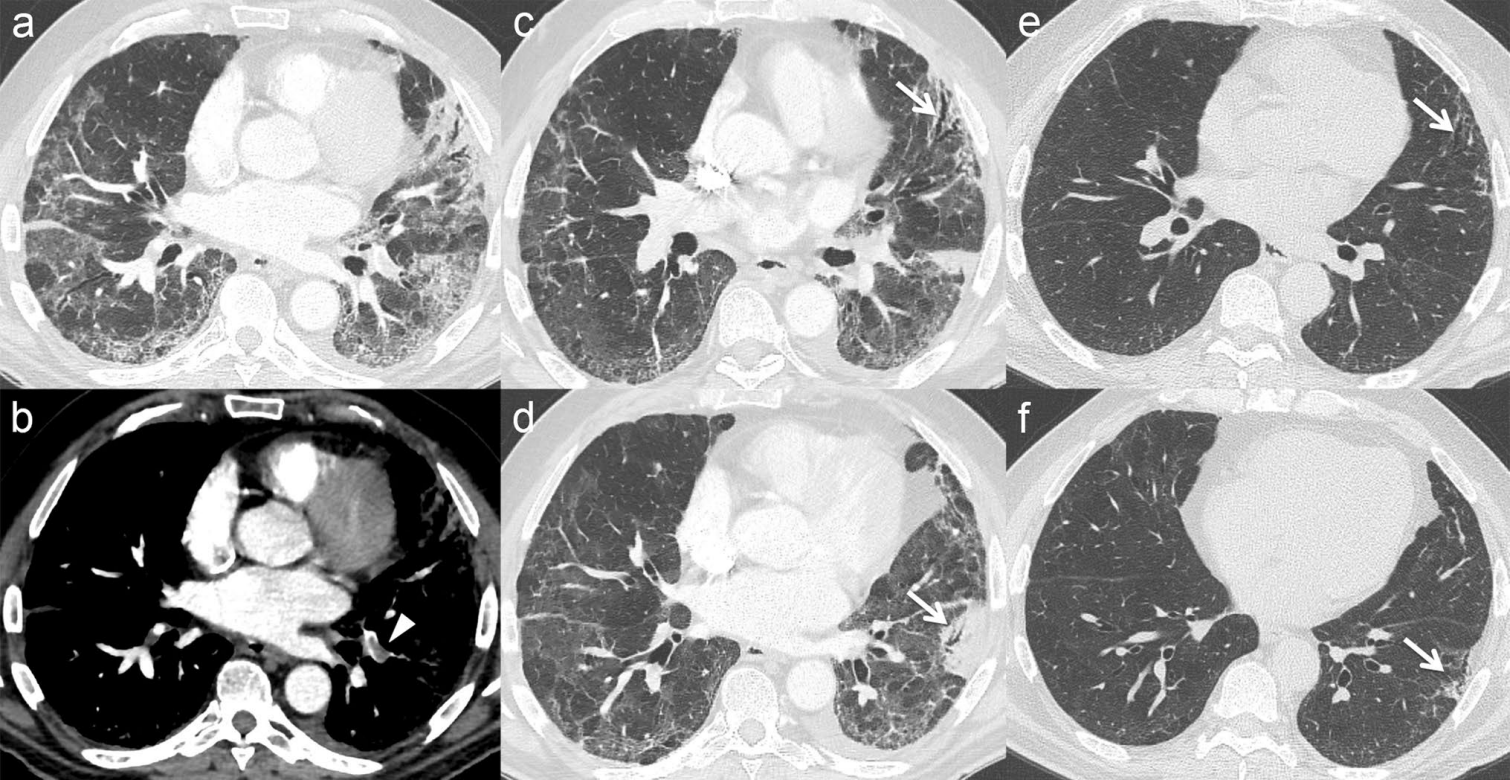
Axial CT scans of a COVID-19 patient with acute pulmonary embolism (arrowhead in **b**), showing the temporal evolution of the lung abnormalities till the late phase. CT scans in **a** and **b** were performed 16 days after the onset of symptoms; CT scans in **c** and **d** 30 days after; CT scans in **e** and **f** 45 days after. Note the progressive reduction of the areas of consolidation and GG—more extensive in the left lung—and the appearance of fibrotic changes with traction bronchiectasis/bronchiolectasis and irregular interstitial thickening in the lingula (arrows in **c** and **e**) and in the left lower lobe (arrow in **f**), mainly in the area of the previously detected lung infarction (arrow in **d**)

HRCT is the imaging modality of choice in the assessment of fibrotic changes [55]. In a study by Wei et al., 39% of patients developed signs of fibrosis on HCRT after recovering from COVID-19 pneumonia [56]. The fibrotic abnormalities described during the late evaluation of COVID-19 pneumonia include irregular interstitial thickening, coarse reticulation, traction bronchiectasis/bronchiolectasis, irregular interfaces, and parenchymal bands [57] (Fig. 5). However, some of these features, especially irregular interfaces and parenchymal bands, may be gradually absorbed over time [57].

The development of fibrotic abnormalities correlates with older age, longer hospitalization, severe disease with ICU admission, and a higher extend of parenchymal abnormalities on HRCT [56, 57]. These evidences have been confirmed in a recently published study by Han X et al., in which 35% (40/114) of patients experiencing a severe form of COVID-19 pneumonia showed fibrotic changes (namely traction bronchiectasis, fibrotic bands and honeycombing) in the follow-up CT scan performed 6 months after the disease onset [58]. The development of fibrotic abnormalities was associated with older age, non-invasive mechanical ventilation requirement, ARDS, longer hospitalization, tachycardia, extensive parenchymal involvement on initial chest CT, and carbon monoxide diffusion lung capacity (DLCO) impairment [58].

Apart from fibrotic abnormalities, other findings can be observed on the CT scans of patients in follow-up after COVID-19 pneumonia. These include the development of new areas of cavitation and/or emphysematous changes 3 months after discharge, reported in 25% of surviving patients who required mechanical ventilation [59].

Nonetheless, considering the multi-organ involvement of COVID-19 and due to the unknown possible evolution and sequelae even in patients fully recovered [60], a multidisciplinary post-acute and late approach becomes mandatory to define clinical needs and ensure a global evaluation [53].

## Prognostic implications of imaging findings

Chest imaging plays a fundamental role in patients with COVID-19 pneumonia not only for assessing the presence and evolution of pulmonary abnormalities but also for defining the severity of disease and predicting patient outcome.

The pivotal role of chest imaging in COVID-19 patients has been evaluated and confirmed in numerous studies; some of them aimed at correlating imaging findings with clinical features and the severity of disease, whereas others focused on the correlation between imaging findings at admission (or in the emergency department) and patients’ outcome to identify factors predictive of disease progression, ICU admission, or death. A common limitation of these studies is their retrospective design, even though they usually involved a re-reading of imaging exams by expert radiologists blinded to patients’ conditions and outcome. Furthermore, the impact of treatment or confounding logistical factors due to health care overload in epidemic areas could not be assessed. Nevertheless, these studies provide evidence on the role of chest imaging for both assessment of severity of disease and prediction of outcome in the various clinical conditions of COVID-19 patients.

### Chest imaging as an indicator of disease severity and patient outcome

The distribution, extent, and type of pulmonary abnormalities as seen on thin-section CT scans of the chest correlate with the severity of COVID-19 pneumonia [45, 61–64]. In general, patients with severe or critical disease show bilateral lung involvement and a more diffuse distribution of pulmonary abnormalities that tend to extend from the lower to the upper lobes [45, 62, 65] and from the peripheral to the parahilar zones of the lung [62, 64]. Furthermore, areas of consolidation, *crazy paving*, air bronchogram, and bronchial wall thickening have been reported more frequently in severe/critical patients than in those with a mild course of the disease [45, 61, 64].

When a CT severity score based on semi-quantitative assessment of the extent of pulmonary abnormalities is used, patients with severe/critical disease show significantly higher scores than patients with a mild disease [45, 61–63].

A severity score helps in quantifying the pulmonary involvement and has been used in other clinical conditions such as pulmonary edema and acute respiratory infection in hospitalized patients [66, 67]. Different scoring systems have been tested in various studies including COVID-19 patients and have resulted in a good correlation with the severity of disease and good inter-reader agreement. A few examples are included in the present review.

In one study of 102 COVID-19 patients, including 84 with mild disease and 18 with severe disease, the CT severity score was defined as the sum of individual scores from 20 lung regions; scores of 0, 1, and 2 were assigned to each region if parenchymal opacification involved 0%, less than 50%, or equal or more than 50% of that region, respectively [63]. Study results showed a significantly higher severity score in patients with severe COVID-19 disease than in patients with mild disease (median scores of 23.5 and 13.0, respectively; *P* < 0.001). The optimal score threshold for identifying severe patients was 19.5, with 83.3% sensitivity and 94% specificity. Inter-observer agreement was excellent between the two radiologists who retrospectively assessed the CT scans.

In the study by Li et al., clinical data and CT findings of 83 patients with COVID-19 pneumonia (25 severe/critical patients and 58 ordinary patients) were reviewed and compared [45]. To quantify the extent of lesions on CT scans, each pulmonary lobe was assigned a score of 0–5 on the basis of the percentage of involvement (0%, less than 5%, 5–25%, 26–49%, 50–75%, and greater than 75%); a total score was then calculated. A software was applied to obtain 3D visualization by automatically segmenting the whole lung and pulmonary lesions. Similar to the previously described studies, the CT severity score in severe/critical patients was significantly higher than the score in ordinary patients (median score, 11 vs 5; *P* < 0.001). When the cutoff value of CT score was 7, the sensitivity and specificity for the ordinary versus severe/critical patients discrimination were 80.0% and 82.8%, respectively.

**Fig. 6:**
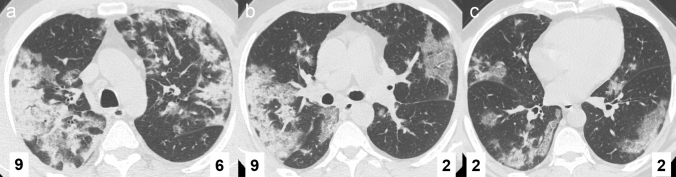
HRCT scans from the upper (**a**), middle (**b**), and lower (**c**) zones in a 54-year-old man who developed a severe course of COVID-19 pneumonia. The total CT severity score is 30, based on the scoring system proposed by Yuan et al. [64]: **a** right upper, 3 (consolidation) × 3 (extent 50–75%); left upper, 3 (consolidation) × 2 (25–50%); **b** right middle, 3 (consolidation) × 3 (50–75%); left middle, 2 (ground-glass) × 1 (< 25%); **c** right lower, 2 (ground-glass) × 1 (< 25%); left lower, 2 (ground-glass) × 1 (< 25%)

A different approach for the definition of the CT severity score was used by Yuan et al. [64]. Each lung is divided into 3 zones: upper (above the carina), middle (between the carina and the inferior pulmonary vein), and lower (below the pulmonary vein). Each lung zone is assigned a lesion score (1 as normal attenuation; 2 as GG attenuation; 3 as consolidation) and an abnormality extent score (0 as normal; 1 as less than 25% abnormality; 2 as 25–50% abnormality; 3 as 50–75% abnormality; 4 as greater than 75% abnormality). The two scores are then multiplied to obtain the score for each zone; the sum of the 6 lung zones provides the total cumulative CT severity score (Fig. 6). In a validation study of 27 patients with COVID-19 pneumonia, the median CT score was significantly higher among patients who died than among those who recovered (30 vs 12; *P* = 0.021). In the ROC analysis, an optimal cutoff value of 24.5 for the CT score had a sensitivity of 85.6% and a specificity of 84.5% for the prediction of mortality.

Quantitative analyses of lung abnormalities on baseline CT scans can correlate with clinical course in patients with COVID-19 pneumonia and could be effectively used in association with clinical and laboratory features to predict patient outcome. Numerous studies have been published in the last few months demonstrating that CT findings at admission and their quantification help predict in-hospital mortality [68], risk for ICU admission [65, 69], or progression to severe illness [70, 71]. In most cases, a deep-learning-based quantitative CT imaging was used for automatic segmentation and quantification of the extent of lung involvement [65, 68–70].

In one study, the extent of well-aerated lung at baseline chest CT performed in the emergency department was assessed and quantified to determine its relationship with prognosis [68]. Patients with COVID-19 pneumonia who were admitted to ICU or died showed involvement of 4 or more lobes at baseline chest CT more frequently than patients without ICU admission or death (16% versus 6% of patients, *P* < 0.04). After adjustment for patient demographics and clinical parameters, visually assessed well-aerated lung parenchyma on admission CT less than 73% was an independent predictor of ICU admission or death (odds ratio 5.4, *P* < 0.001); software-based quantification of the extent of well-aerated lung showed similar results. As described by the authors, CT quantification of well-aerated lung was shown to help estimating the alveolar recruitment during ventilation and to predict the prognosis of patients with ARDS [72, 73]. Quantification of the extent of well-aerated lung, rather than of parenchymal abnormalities, would avoid including underlying lung diseases, such as emphysema or fibrosis, in the estimate; it could also represent a surrogate of residual respiratory function [68].

Although the sensitivity of CXR for diagnosis of COVID-19 is relatively low, the utility of initial CXR on predicting clinical outcome represents an unmet need, especially in a clinical context with a high influx of patients [74].

The value of the initial CXR in young adults (21–50 years) with a confirmed diagnosis of COVID-19 was reported by Toussie et al. using a very simple scoring system [74]. Each lung was divided into 3 zones (lower from the costophrenic sulcus to inferior hilar markings, middle from inferior hilar markings to superior hilar markings, upper from superior hilar markings to the apices). Each zone was given a binary score, depending on the presence (1) or absence (0) of opacities, which were then summed for a total score (0–6). This scoring system was tested in a retrospective study of 338 patients who presented to the emergency department at a hospital in NYC [74]. After adjusting for demographics and co-morbidities, the severity of opacification on the initial CXR was associated with the need for hospitalization and need for intubation. Patients with opacities in at least 2 lung zones were more likely to require hospitalization and those with opacities in at least 3 lung zones were more likely to require intubation. The authors also found that age, male gender, and obesity were associated with an increased risk of a higher CXR score (≥ 2) and need for hospitalization and intubation.

**Fig. 7:**
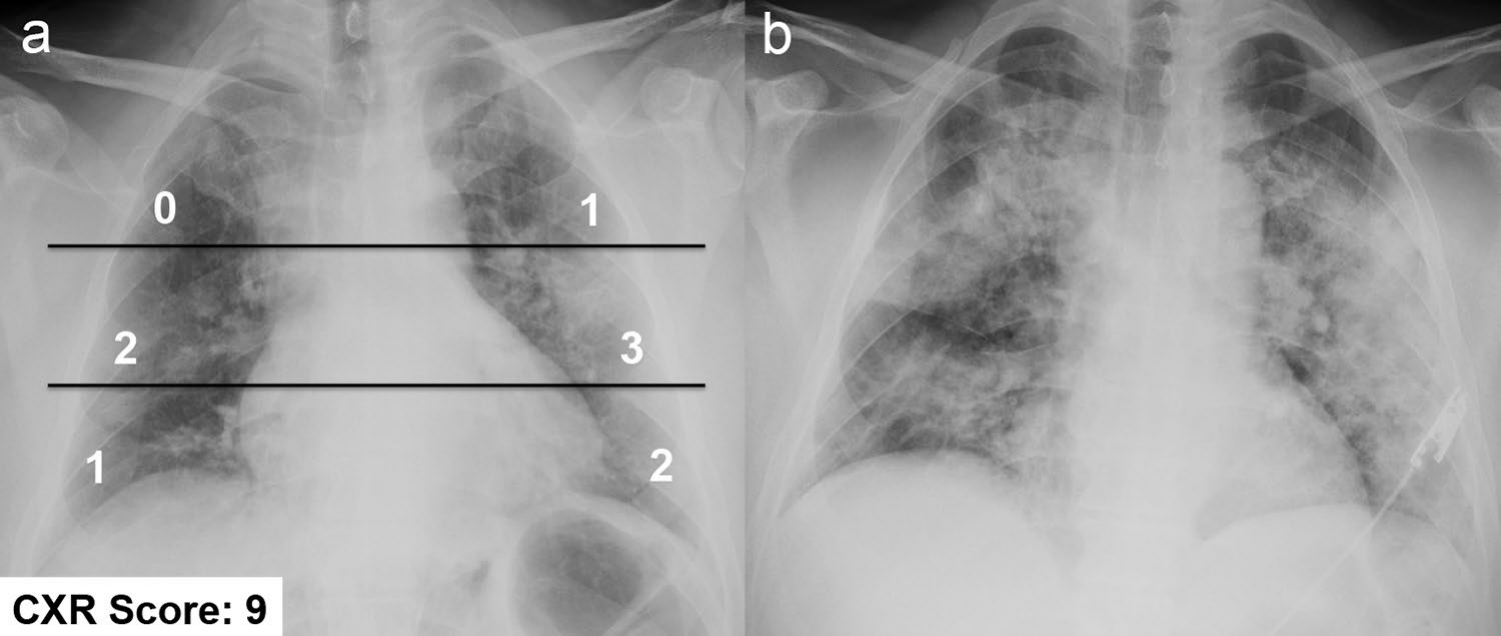
Bedside chest X-ray performed at the emergency department admission in a 62-years-old male affected by COVID-19 pneumonia (**a**), showing a Brixia score of 9 [67]. Note that the upper line is drawn at the level of the inferior wall of the aortic arch, while the lower line is drawn at the level of the inferior wall of the right inferior pulmonary vein. Based on the chest X-ray severity score (≥ 8) and age (≥ 50 years-old), this patient had an increased risk of developing a severe disease course. Indeed, the chest X-ray performed 6 days later (**b**) depicts a clear disease progression, with increase in density and extent of the abnormalities in both lungs. Lately, the patient recovered after ICU admission

Three studies from the same site in Italy correlated CXR findings at admission with patients’ outcome to identify in-hospital mortality; all patients had a confirmed diagnosis of COVID-19 [67, 75, 76]. The authors designed a dedicated scoring system (called Brixia score) for semi-quantitative assessment of lung disease in COVID-19 patients based on the extent and characteristics of lung abnormalities [67]. Each of 6 lung zones (upper, middle, inferior) is assigned a score as follows: 0 to indicate the absence of lung abnormalities, 1 if interstitial infiltrates are present, 2 if interstitial and alveolar infiltrates are present with a predominance of interstitial opacities, 3 if interstitial and alveolar infiltrates are present with a predominance of alveolar opacities. The scores of each lung zone are then added to obtain an overall CXR score (0–18) (Fig. 7). In the multivariable logistic regression model, Brixia score, patient age, and conditions that induced immunosuppression were the only significant predictive factors for in-hospital mortality. The optimal cutoff values for Brixia score and patient age were 8 points and 71 years, respectively [75]. The authors also found that males ≥ 50 years and females ≥ 80 years showed the highest score (median ≥ 8) and were at higher risk for developing severe lung disease [76].

### Other chest imaging findings associated with prognosis

Other chest CT findings that seem to be associated with prognosis of patients with COVID-19 include pulmonary embolism (PE) and enlargement of mediastinal lymph nodes.

Thrombotic complications, including PE, are emerging as important sequelae that contribute to significant morbidity and mortality in patients diagnosed with COVID-19 [71].

In recently published studies, the incidence of PE among hospitalized COVID-19 patients who underwent CT angiography ranged between 21 and 30% [13, 77–80]. This incidence is higher than that reported in critically ill patients without COVID-19 infection (1.3%) or in the emergency department (3–10%) [13]. In most studies, patients with PE were more frequently in the ICU, required mechanical ventilation more often, and had a longer delay between onset of symptoms and CT scan than patients without PE [13, 78, 79]. They also showed higher levels of D-dimer. In one study, a significant difference in C-reactive protein and D-dimer between PE positive and PE negative patients was observed, which may suggest that COVID-19 positive patients with higher levels of inflammation and D-dimer values are more susceptible to developing pulmonary embolism [77].

Acute PE was also described in 13 of 72 (18%) non-hospitalized COVID-19 patients, referred to CT angiography from the emergency department [14]. In this study, patients with PE were older and had higher D-dimer levels than patients without PE, whereas no significant difference was found in the severity of pulmonary involvement at CT.

Given the high incidence of PE in patients with COVID-19 pneumonia, some authors suggested a larger use of contrast-enhanced CT in these patients, especially for those with marked elevation of D-dimer [14, 78, 79]. According to the advice paper from the European Society of Radiology and the European Society of Thoracic Imaging, contrast-enhanced CT may be indicated to rule out PE if supplementary oxygen is needed in COVID-19 patients with limited disease extension [6].

Recent reports have highlighted the relatively high number of COVID-19 patients showing mediastinal lymphadenopathies larger than 10 mm in short axis to the point that the authors questioned the initial classification of lymphadenopathies as an atypical feature of COVID-19 [81, 82]. In a cohort of 410 COVID-19 patients who underwent CT at the time of admission to the emergency department, Sardanelli et al. found a prevalence of mediastinal lymph node enlargement of 19%, higher than the pooled prevalence previously reported in two systematic reviews (3.4% and 5.4%) [31, 83]. The prevalence increased to 27% in those patients who died during hospitalization, indicating that mediastinal lymphadenopathies may be considered a predictor of a worse outcome [81]. In a smaller cohort of 15 patients in the ICU, the prevalence of mediastinal lymphadenopathies was even higher (66%) [82].

## Conclusions

Chest imaging plays a crucial role in the initial evaluation and follow-up of patients with COVID-19 pneumonia. Chest imaging can also help in the assessment of disease severity and prediction of patient outcome, either as an independent factor or in combination with clinical and laboratory features.

## References

[1] Huang C, Wang Y, Li X (2020). Clinical features of patients infected with 2019 novel coronavirus in Wuhan, China. Lancet.

[2] Pan A, Liu L, Wang C (2020). Association of public health interventions with the epidemiology of the COVID-19 outbreak in Wuhan, China. JAMA.

[3] Guan WJ, Ni ZY, Hu Y (2020). Clinical characteristics of coronavirus disease 2019 in China. N Engl J Med.

[4] Quah P, Li A, Phua J (2020). Mortality rates of patients with COVID-19 in the intensive care unit: a systematic review of the emerging literature. Crit Care..

[5] Hani C, Trieu NH, Saab I (2020). COVID-19 pneumonia: a review of typical CT findings and differential diagnosis. DiagnInterv Imaging.

[6] Revel MP, Parkar AP, Prosch H (2020). COVID-19 patients and the radiology department—advice from the European Society of Radiology (ESR) and the European Society of Thoracic Imaging (ESTI). EurRadiol.

[7] Rubin GD, Ryerson CJ, Haramati LB (2020). The role of chest imaging in patient management during the COVID-19 pandemic: a multinational consensus statement from the Fleischner Society. Chest.

[8] Larici AR, Cicchetti G, Marano R (2020). Multimodality imaging of COVID-19 pneumonia: from diagnosis to follow-up. A comprehensive review. Eur J Radiol.

[9] McGuinness G, Zhan C, Rosenberg N (2020). Increased incidence of barotrauma in patients with COVID-19 on invasive mechanical ventilation. Radiology.

[10] Rawson TM, Moore LSP, Zhu N (2020). Bacterial and fungal coinfection in individuals with coronavirus: a rapid review to support COVID-19 antimicrobial prescribing. Clin Infect Dis.

[11] Bikdeli B, Madhavan MV, Jimenez D (2020). COVID-19 and thrombotic or thromboembolic disease: implications for prevention, antithrombotic therapy, and follow-up. J Am Coll Cardiol.

[12] Magro C, Mulvey JJ, Berlin D (2020). Complement associated microvascular injury and thrombosis in the pathogenesis of severe COVID-19 infection: a report of five cases. Transl Res.

[13] Leonard-Lorant I, Delabranche X, François Severac F (2020). Acute pulmonary embolism in COVID-19 patients on CT angiography and relationship to D-dimer levels. Radiology.

[14] Gervaise A, Bouzad C, Peroux E, Helissey C (2020). Acute pulmonary embolism in non-hospitalized COVID-19 patients referred to CTPA by emergency department. Eur Radiol.

[15] Xu Z, Shi L, Wang Y (2020). Pathological findings of COVID-19 associated with acute respiratory distress syndrome. Lancet Respir Med.

[16] Schaller T, Hirschbühl K, Burkhardt K (2020). Postmortem examination of patients with COVID-19. JAMA.

[17] Carsana L, Sonzogni A, Ahmed Nasr A (2020). Pulmonary post-mortem findings in a series of COVID-19 cases from Northern Italy: a two-centre descriptive study. Lancet Infect Dis.

[18] Ichikado K (2014). High-resolution computed tomography findings of acute respiratory distress syndrome, acute interstitial pneumonia, and acute exacerbation of idiopathic pulmonary fibrosis. Semin Ultrasound CT MR.

[19] Ichikado K, Suga M, Gushima Y (2000). Hyperoxia-induced diffuse alveolar damage in pigs: correlation between thin-section CT and histopathologic findings. Radiology.

[20] Pan F, Ye T, Sun P (2020). Time course of lung changes at chest CT during recovery from 2019 novel Coronavirus (COVID-19) pneumonia. Radiology.

[21] Wang Y, Dong C, Hu Y (2020). Temporal changes of CT findings in 90 patients with COVID-19 pneumonia: a longitudinal study. Radiology.

[22] Wong HYF, Lam HYS, Fong AH (2020). Frequency and distribution of chest radiographic findings in COVID-19 positive patients. Radiology.

[23] Vancheri SG, Savietto G, Ballati F (2020). Radiographic findings in 240 patients with COVID-19 pneumonia: time-dependence after the onset of symptoms. EurRadiol.

[24] Ojha V, Mani A, Pandey NN (2020). CT in Coronavirus disease 2019 (COVID-19): a systematic review of chest CT findings in 4410 adult patients. EurRadiol.

[25] Spagnolo P, Balestro E, Aliberti S (2020). Pulmonary fibrosis secondary to COVID-19: a call to arms?. Lancet Respir Med.

[26] George PM, Wells AU, Jenkins RG (2020). Pulmonary fibrosis and COVID-19: the potential role for antifibrotic therapy. Lancet Respir Med.

[27] Shi H, Han X, Jiang N (2020). Radiological findings from 81 patients with COVID-19 pneumonia in Wuhan, China: a descriptive study. Lancet Infect Dis.

[28] Han X, Cao Y, Jiang N (2020). Novel Coronavirus pneumonia (COVID-19) progression course in 17 discharged patients: comparison of clinical and thin-Section CT features during recovery. Clin Infect Dis.

[29] Zhou X, Pu Y, Zhang D (2021). CT findings and dynamic imaging changes of COVID-19 in 2908 patients: a systematic review and meta-analysis. Acta Radiol..

[30] Salehi S, Abedi A, Balakrishnan S, Gholamrezanezhad A (2020). Coronavirus disease 2019 (COVID-19): a systematic review of imaging findings in 919 patients. AJR Am J Roentgenol.

[31] Bao C, Liu X, Zhang H (2020). Coronavirus disease 2019 (COVID-19) CT findings: a systematic review and meta-analysis. J Am Coll Radiol.

[32] Adams HJA, Kwee TC, Yakar D (2020). Chest CT imaging signature of coronavirus disease 2019 infection: in pursuit of the scientific evidence. Chest.

[33] Xie X, Zhong Z, Zhao W (2020). Chest CT for typical 2019-nCoV pneumonia: relationship to negative RT-PCR testing. Radiology.

[34] Ye Z, Zhang Y, Wang Y (2020). Chest CT manifestations of new coronavirus disease 2019 (COVID-19): a pictorial review. EurRadiol.

[35] Caruso D, Zerunian M, Polici M (2020). Chest CT features of COVID-19 in Rome, Italy. Radiology.

[36] Li X, Zeng W, Li X (2020). CT Imaging changes of Corona virus disease 2019(COVID-19): a multi-center study in Southwest China. J Transl Med.

[37] Li Y, Xia LM (2020). Coronavirus disease 2019 (COVID-19): role of chest CT in diagnosis and management. AJR Am J Roentgenol.

[38] Ye Q, Wang B, Mao J (2020). The pathogenesis and treatment of the ‘cytokine storm' in COVID-19. J Infect.

[39] Bai HX, Hsieh B, Xiong Z (2020). Performance of radiologists in differentiating COVID-19 from viral pneumonia on chest CT. Radiology.

[40] Ding X, Xu J, Zhou J, Long Q (2020). Chest CT findings of COVID-19 pneumonia by duration of symptoms. Eur J Radiol.

[41] Bernheim A, Mei X, Huang M (2020). Chest CT findings in Coronavirus disease-19 (COVID-19): relationship to duration of infection. Radiology.

[42] Hu Q, Guan H, Sun Z (2020). Early CT features and temporal lung changes in COVID-19 pneumonia in Wuhan, China. Eur J Radiol.

[43] Zhou S, Zhu T, Wang Y, Xia LM (2020). Imaging features and evolution on CT in 100 COVID-19 pneumonia patients in Wuhan, China. EurRadiol.

[44] Wu C, Chen X, Cai Y (2020). Risk factors associated with acute respiratory distress syndrome and death in patients with coronavirus disease 2019 pneumonia in Wuhan, China. JAMA Intern Med.

[45] Li K, Wu J, Wu F (2020). The clinical and chest CT features associated with severe and critical COVID-19 pneumonia. InvestigRadiol.

[46] Hansell DM, Bankier AA, MacMahon H (2008). Fleischner Society: glossary of terms for thoracic imaging. Radiology.

[47] Kligerman SJ, Franks TJ, Galvin JR (2013). From the radiologic pathology archives: organization and fibrosis as a response to lung injury in diffuse alveolar damage, organizing pneumonia, and acute fibrinous and organizing pneumonia. Radiographics.

[48] Tian S, Hu W, Niu L (2020). Pulmonary pathology of early-phase 2019 novel Coronavirus (COVID-19) pneumonia in two patients with lung cancer. J Thorac Oncol.

[49] Wu Y, Xie Y, Wang X (2020). Longitudinal CT findings in COVID-19 pneumonia: case presenting organizing pneumonia pattern. RadiolCardiothoracImaging.

[50] Liu D, Zhang W, Pan F (2020). The pulmonary sequalae in discharged patients with COVID-19: a short-term observational study. Respir Res.

[51] You J, Zhang L, Ti MN (2020). Anormal pulmonary function and residual CT abnormalities in rehabilitating COVID-19 patients after discharge. J Infect.

[52] Huang G, Gong T, Wang G (2020). Timely diagnosis and treatment shortens the time to resolution of Coronavirus disease (COVID-19) pneumonia and lowers the highest and last CT scores from sequential chest CT. AJR Am J Roentgenol.

[53] Landi F, Gremese E, Bernabei R (2020). Post-COVID-19 global health strategies: the need for an interdisciplinary approach. Aging ClinExp Res.

[54] Mo X, Jian W, Su Z (2020). Abnormal pulmonary function in COVID-19 patients at time of hospital discharge. EurRespir J.

[55] Sundaram B, Chughtai AR, Kazerooni EA (2010). Multidetector high-resolution computed tomography of the lungs: protocols and applications. J Thorac Imaging.

[56] Wei J, Yang H, Lei P (2020). Analysis of thin-section CT in patients with Coronavirus disease (COVID-19) after hospital discharge. J Xray Sci Technol.

[57] Yu M, Liu Y, Xu D (2020). Prediction of the development of pulmonary fibrosis using serial thin-section CT and clinical features in patients discharged after treatment for COVID-19 pneumonia. Korean J Radiol.

[58] Han X, Fan Y, Alwalid O (2021). Six-month follow-up chest CT findings after severe COVID-19 pneumonia. Radiology.

[59] van Gassel RJJ, Bels JLM, Raafs A (2021). High prevalence of pulmonary sequelae at 3 months after hospital discharge in mechanically ventilated survivors of COVID-19. Am J Respir Crit Care Med.

[60] Kanne JP, Bai H, Bernheim A (2021). COVID-19 imaging: what we know now and what remains unknown. Radiology.

[61] Zhang R, Ouyang H, Fu L (2020). CT features of SARS-CoV-2 pneumonia according to clinical presentation: a retrospective analysis of 120 consecutive patients from Wuhan city. EurRadiol.

[62] Zhang N, Xu X, Zhou L-Y (2020). Clinical characteristics and chest CT imaging features of critically ill COVID-19 patients. EurRadiol.

[63] Yang R, Li X, Liu H (2020). Chest CT severity score: an imaging tool for assessing severe COVID-19. RadiolCardiothorac Imaging.

[64] Yuan M, Yin W, Tao Z, Tan W, Hu Y (2020). Association of radiologic findings with mortality of patients infected with 2019 novel coronavirus in Wuhan, China. PLoS ONE.

[65] Burian E, Jungmann F, Kaissis GA (2020). Intensive care risk estimation in COVID-19 pneumonia based on clinical and imaging parameters: experiences from the Munich cohort. J Clin Med.

[66] Cozzi D, Albanesi M, Cavigli E (2020). Chest X-ray in new coronavirus disease 2019 (COVID-19) infection: findings and correlation with clinical outcome. Radiol Med.

[67] Borghesi A, Maroldi R (2020). COVID-19 outbreak in Italy: experimental chest X-ray scoring system for quantifying and monitoring disease progression. Radiol Med.

[68] Colombi D, Bodini FC, Petrini M (2020). Well-aerated lung on admitting chest CT to predict adverse outcome in COVID-19 pneumonia. Radiology.

[69] Yu Q, Wang Y, Huang S (2020). Multicenter cohort study demonstrates more consolidation in upper lungs on initial CT increases the risk of adverse clinical outcome in COVID-19 patients. Theranostics.

[70] Liu F, Zhang Q, Huang C (2020). CT quantification of pneumonia lesions in early days predicts progression to severe illness in a cohort of COVID-19 patients. Theranostics.

[71] Liu Z, Jin C, Wu CC (2020). Association between initial chest CT or clinical features and clinical course in patients with coronavirus disease 2019 pneumonia. Korean J Radiol.

[72] Gattinoni L, Caironi P, Cressoni M (2006). Lung recruitment in patients with the acute respiratory distress syndrome. N Engl J Med.

[73] Nishiyama A, Kawata N, Yokota H (2020). A predictive factor for patients with acute respiratory distress syndrome: CT lung volumetry of well-aerated region as an automated method. Eur J Radiol.

[74] Toussie D, Voutsinas N, Finkelstein M (2020). Clinical and chest radiography features determine patient outcomes in young and middle age adults with COVID-19. Radiology.

[75] Borghesi A, Zigliani A, Golemi S (2020). Chest X-ray severity index as a predictor of in-hospital mortality in coronavirus disease 2019: a study of 302 patients from Italy. Int J Infect Dis.

[76] Borghesi A, Zigliani A, Masciullo R (2020). Radiographic severity index in COVID-19 pneumonia: relationship to age and sex in 783 Italian patients. Radiol Med.

[77] Poyiadi N, Cornier P, Patel PY (2020). Acute pulmonary embolism and COVID-19. Radiology.

[78] Bompard F, Monnier H, Saab I (2020). Pulmonary embolism in patients with COVID-19 pneumonia. EurRespir J.

[79] Grillet F, Behr J, Calame P, Aubry S, Delabrousse E (2020). Acute pulmonary embolism associated with COVID-19 pneumonia detected by pulmonary CT angiography. Radiology.

[80] Poissy J, Goutay J, Capln M (2020). Pulmonary embolism in COVID-19 patients: awareness of an increased prevalence. Circulation.

[81] Sardanelli F, Cozzi A, Monfardini L (2020). Association of mediastinal lymphadenopathy with COVID-19 prognosis. Lancet Infect Dis.

[82] Valette X, du Cheyron D, Goursaud S (2020). Mediastinal lymphadenopathy in patients with severe COVID-19. Lancet Infect Dis.

[83] Zhu J, Zhong Z, Li H (2020). CT imaging features of 4121 patients with COVID-19: a meta-analysis. J Med Virol.

